# A Palette of Fluorescent Thiophene-Based Ligands for the Identification of Protein Aggregates

**DOI:** 10.1002/chem.201502999

**Published:** 2015-09-21

**Authors:** Hamid Shirani, Mathieu Linares, Christina J Sigurdson, Mikael Lindgren, Patrick Norman, K Peter R Nilsson

**Affiliations:** [a] Division of Chemistry, Department of Physics, Chemistry and Biology, Linköping University 581 83 Linköping (Sweden) E-mail: petni@ifm.liu.se; [b] Division of Theoretical Chemistry, Department of Physics, Chemistry and Biology, Linköping University 581 83 Linköping (Sweden); [c] Department of Pathology, University of California San Diego, La Jolla, California 92093-0612 (USA)

**Keywords:** Alzheimer’s disease, fluorescent probes, luminescence, oligothiophenes, microscopy

## Abstract

By replacing the central thiophene unit of an anionic pentameric oligothiophene with other heterocyclic moities, a palette of pentameric thiophene-based ligands with distinct fluorescent properties were synthesized. All ligands displayed superior selectivity towards recombinant amyloid fibrils as well as disease-associated protein aggregates in tissue sections.

Small fluorescent ligands are essential for visualizing protein aggregates, the common pathological hallmark of many neurodegenerative diseases such as Alzheimer’s and Parkinson’s disease (AD and PD).[1, 2] In this regard, a variety of molecular scaffolds targeting the regular cross β-pleated sheet conformation of the protein aggregates have been developed.[3–8] Lately, luminescent conjugated poly- and oligothiophenes (LCPs and LCOs) have also been employed as novel tools for fluorescence imaging of protein aggregates and in comparison to conventional ligands, LCOs have been shown to detect a wider range of disease-associated protein aggregates.[9–13] Due to their electronically delocalized conjugated thiophene backbones, LCOs exhibit specific intrinsic fluorescence characteristics and offer the possibility to use a variety of imaging techniques, as well as different modes of detection, such as full excitation/emission spectra, and fluorescence decay time.[14] However, the fluorescent characteristics of LCOs are to some extent restricted and it is of great interest to develop thiophene-based ligands covering a wider range of emission in the visual spectrum. Such fluorescent ligands will be essential in order to design multiplex detection methodologies involving a combination of LCOs as well as fluorophore-labelled antibodies towards distinct proteins.

Herein, we present the synthesis and characterization of a palette of anionic pentameric oligothiophene derivatives and evaluated them as fluorescent ligands for protein aggregates. To achieve a palette of ligands, we started with a previously reported mono-borylated di-thiophene building block[12] (Scheme 1). By applying general synthetic routes, including Suzuki cross-coupling of this building block with di-brominated phenylene, selenophene, quinoxaline, or benzodithiazole, followed by de-protection of the anionic side chains, four pentameric thiohene-based ligands, HS-163, HS-165, HS-167, and HS-169 with distinct central heterocyclic moieties were synthesized. In addition, the previously reported pentameric oligothiophene, HS-84 (Scheme 1),[13] was included in the study.

**Scheme 1:**
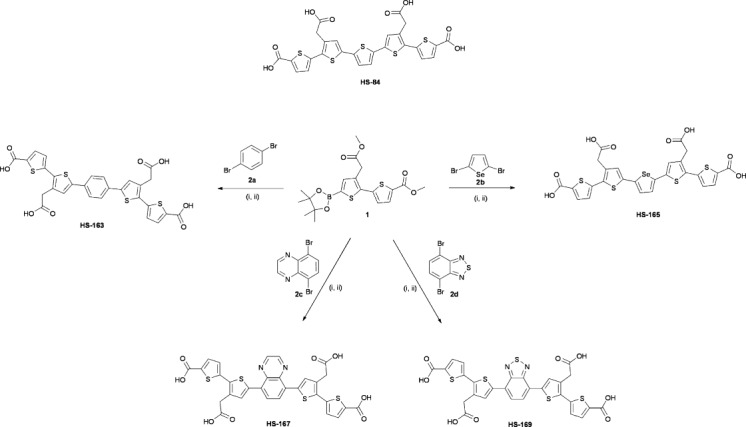
Synthesis of HS-163, HS-165, HS-167, and HS-169. Reagents and conditions: i) 1,4-dioxane/MeOH, PEPPSI™-IPr, K_2_CO_3_, 70 °C, 20 min; ii) NaOH (1 m), 1,4-dioxane, 60 °C, 16 h. The synthesis of compound 1 and HS-84 has been reported elsewhere.[12, 13] PEPPSI=pyridine-enhanced precatalyst preparation stabilization and initiation.

When diluted in phosphate-buffered saline (PBS, pH 7.4) all the novel ligands displayed different absorption characteristics compared to HS-84 (Figure 1 A). HS-163 showed a blueshift of the absorption maximum, whereas the absorption maximum of HS-165 was slightly redshifted. The slight redshift for HS-165 might originate from an increased quinoid character of the central selenophene giving the inter-ring C–C bond more double-bond properties and an enhanced efficiency of conjugation through the backbone.[15–17] In a similar fashion, the central phenyl moiety will disrupt the planarity and the conjugation length, leading to a blueshifted absorption maximum for HS-163 compared to HS-84.[18–20] HS-167 and HS-169, which have donor–acceptor–donor (d-A-D)-type electronic structure, displayed two absorption maxima (Figure 1 A). This approach, utilizing nitrogen-containing heterocycles as effective electron acceptors and thiophenes as donors, has been reported previously.[21, 22] The two absorbance bands, a high energy band around 360 nm, followed by a low energy band at 460 (HS-167) or 515 nm (HS-169), likely arise from the π–π* transition and charge-transfer transition, respectively.

**Figure 1:**
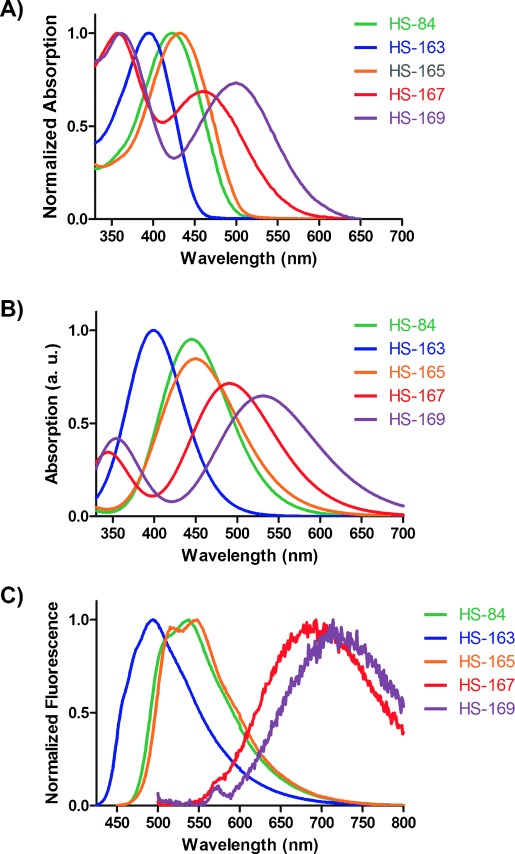
Experimental (A) and calculated (B) absorption spectra, and emission spectra (C) of the thiophene-based pentameric ligands.

Calculations of UV absorption spectra were performed on model systems with the –CH2–COOH groups on the two thiophenes surrounding the central unit being replaced by methyl groups (Figure S1 in the Supporting Information). This chromophore modification is denoted with primes in the name labels, so it is to be understood that labels HS′-84, HS′-165, HS′-163, HS′-167, and HS′-169 refer to the model systems employed in the calculations. As expected due to the hydrogen–sulfur steric interactions in oligothiophenes, molecules HS′-84, HS′-165, and HS′-163 are not flat in their optimized structures, showing inter-ring torsional angles between 10.0 and 24.1 degrees (Figure S2 in the Supporting Information). The backbones of molecules HS′-167 and HS′-169, on the other hand, are very flat with inter-ring torsional angles very close to zero. This finding is partly attributed to favorable sulfur–nitrogen non-bonded interactions between sulfur atoms belonging to thiophenes and the nitrogen atoms of the quinoxaline or benzothiadiazole moieties. These interactions explain the small values for dihedral angles *Φ*2 and *Φ*3, but fail to rationalize the equally small values for dihedral angles *Φ*1 and *Φ*4. Calculated UV spectra were in excellent agreement with the experimental counterparts and the main absorption bands for the series of molecules are summarized in Table S1 in the Supporting Information. For HS′-84, HS′-165, and HS′-163, the sole absorption band found in this region is attributed to an electronic transition between the highest occupied (HOMO) to the lowest unoccupied molecular orbital (LUMO). For HS′-167 and HS′-169, the respective main peak is also attributed to a HOMO–LUMO transition, but they are strongly redshifted with respect to the peak for HS′-84 by 46 and 86 nm, respectively. A strong correlation between redshift and planarity in anionic oligothiophene derivatives has been reported and it was demonstrated that shifts in transition wavelengths due to planarity alone can easily reach over 100 nm.[23] For HS′-167 and HS′-169, we also observe the appearance of a second intense band at 344 and 354 nm, respectively. These bands are attributed to combinations of HOMO–LUMO+2 and HOMO-1–LUMO+1 transitions.

In agreement with the absorbance data, the corresponding fluorescence spectra showed similar spectral shifts with emission maxima ranging from 480 nm for HS-163 up to 705 nm for HS-169 (Figure 1 C). The relatively large Stokes shifts displayed for HS-167 and HS-169 (Table S1 in the Supporting Information) are common for π-conjugated donor–acceptor compounds and are most likely attributed to a degree of charge-transfer character.[24, 25] In comparison with HS-84, the selenophene-containing ligand showed a decrease in the fluorescence intensity. This phenomenon has been reported previously in a study with thiophene and selenophene co-polymers and might be dependent on the lowering of the band gap and thereby increasing the probability of non-radiative emission.[26] A decreased intensity of emission was also observed for HS-167 and HS-169, the ligands showing the most redshifted fluorescence. A similar trend, a decrease quantum yield as the fluorescence redshifts, was recently reported for a family of thiophene-based conjugated oligomers.[21]

In order to elucidate selective binding of the ligands to protein aggregates, all five ligands were tested towards amyloid-like fibrils made from recombinant Aβ 1-42 peptide. All of the ligands revealed distinct excitation and emission characteristics when bound to recombinant Aβ fibrils (Figure 2). When mixed with the amyloid-like fibrils, HS-84 (Figure 2 A), HS-163 (Figure 2 B), and HS-165 (Figure 2 C) displayed redshifted excitation spectra with resolved substructures, as well as blueshifted emission spectra with enhanced intensity and the characteristic double peaks reported for anionic oligothiophenes bound to recombinant amyloid-like fibrils.[10–13] HS-167 and HS-169 also display redshifted excitation maxima and blueshifted emission maxima when bound to Aβ 1-42 amyloid-like fibrils (Table S2 in the Supporting Information). In addition, a more pronounced enhancement of the emission intensity was observed from these d-A-D compounds upon interaction with the fibrils (Figure 2 D, E). Thus, similar to the most commonly used amyloid-specific dye, thioflavin T (ThT),[3, 27] HS-167 and 169 indicated a strong increase in fluorescence upon binding to amyloid fibrils. Overall, the five ligands provided distinct optical signatures upon binding to Aβ 1-42 fibrils, verifying that all of the ligands could be utilized for fluorescent assignment of recombinant amyloid-like fibrils.

**Figure 2:**
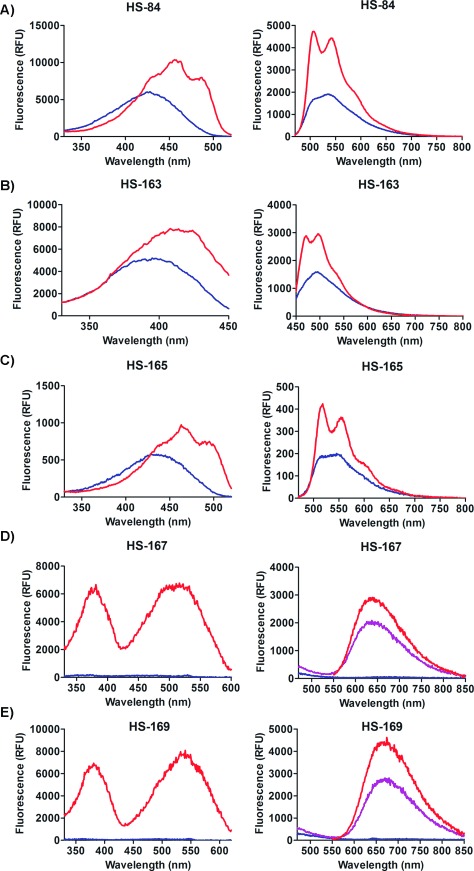
Excitation- (left) and emission (right) spectra of HS-84 (A), HS-163 (B), HS-165 (C), HS-167 (D), and HS-169 (E) in PBS pH 7.4 (blue spectra) or mixed with recombinant Aβ 1-42 amyloid-like fibrils (red spectra). For HS-167 (D) and HS-169 (E) the purple and red emission spectra correlate to excitation at the first (377 nm) or second (510 or 536 nm) excitation maxima, respectively.

Previous studies have shown that pentameric oligothiophenes identify a broader subset of disease-associated protein aggregates than conventional amyloid ligands.[10–12] For instance, two of the major pathological hallmarks of AD, Aβ deposits and tau neurofibrillary tangles (NFTs), have been selectively identified by oligothiophenes in brain tissue sections.[10–12] Therefore, the anionic pentameric oligothiophene derivatives were next evaluated as fluorescent ligands in brain tissue sections with AD pathology. All of the ligands showed specific binding to extracellular Aβ deposits in the brain parenchyma (Aβ core plaques) and in the vasculature (cerebral β-amyloid angiopathy, CAA) (Figure 3). In addition, intracellular NFTs were also stained by all of the ligands (Figure 3). Similar to the observation on recombinant Aβ 1-42 amyloid fibrils, HS-84, HS-163, and HS-165 displayed well-resolved emission spectra with characteristic double peaks upon binding to assemblies of Aβ and tau, whereas HS-167 and HS-169 showed a broad fluorescence spectrum with redshifted emission maxima compared to the other ligands (Figure 3 and Table S2 in the Supporting Information). Thus, from a biological perspective, the three common pathological hallmarks, Aβ core plaques, CAA, and NFTs, could easily be identified due to bright fluorescence as well as distinct spectral signatures from all of the ligands. From a chemical perspective, these experiments also verified that the central thiophene motif could be replaced with other heterocyclic moieties without reducing the ligand′s selectivity towards disease-associated protein aggregates in a complex environment such as tissue sections.

**Figure 3:**
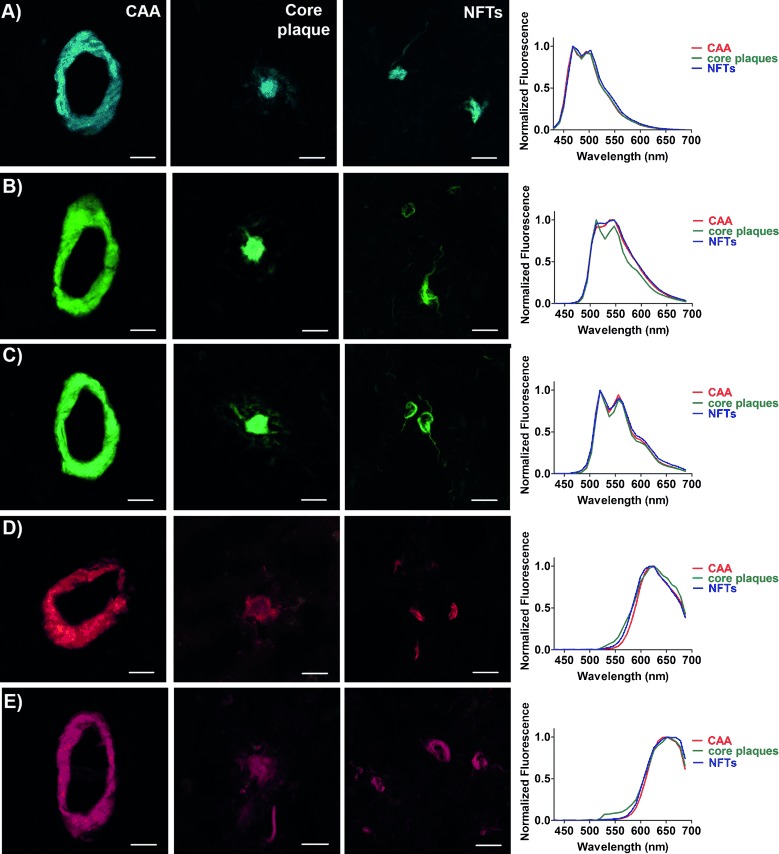
Fluorescence images and emission spectra of HS-163 (A), HS-84 (B), HS-165 (C), HS-167 (D), and HS-169 (E) bound to Aβ deposits in the vasculature (left, CAA) or brain parenchyma (middle, core plaques) and to neurofibrillary tangles (right, NFTs). Scale bars represent 20 μm.

In conclusion, we have demonstrated that a general synthetic route where the central thiophene unit is replaced with other heterocyclic motifs can be used for synthesizing a palette of ligands that can be utilized for fluorescent assessment of protein aggregates. We foresee that these novel thiophene-based pentameric ligands will expand the tool box of fluorescent ligands for identifying a variety of disease-associated protein aggregates, the common pathological hallmarks of several neurodegenerative diseases.

## Supporting Information

As a service to our authors and readers, this journal provides supporting information supplied by the authors. Such materials are peer reviewed and may be re-organized for online delivery, but are not copy-edited or typeset. Technical support issues arising from supporting information (other than missing files) should be addressed to the authors.

miscellaneous_information
