# Supplementary material for: A Palette of Fluorescent Thiophene-Based Ligands for the Identification of Protein Aggregates
Source: Chemistry. 2015 Sep 21;21(43):15133–7. doi: 10.1002/chem.201502999 (PMC4641461; doi:10.1002/chem.201502999)
Supplement: Supplementary file 1 — miscellaneous_information [file chem0021-15133-sd1.pdf]

CHEMISTRY
A European Journal

Supporting Information

# A Palette of Fluorescent Thiophene-Based Ligands for the Identification of Protein Aggregates

Hamid Shirani,[a] Mathieu Linares,[b] Christina J. Sigurdson,[c] Mikael Lindgren,[a] Patrick Norman,[b] and K. Peter R. Nilsson*[a]

chem_201502999_sm_miscellaneous_information.pdf

# Supporting Information

## A palette of thiophene based ligands for fluorescent identification of protein aggregates

*H. Shirani,[a] M. Linares[b], C.J. Sigurdson[c], , M. Lindgren[a,d], P. Norman[b] and K. P. R. Nilsson[a*]*

[a] Division of Chemistry, Department of Physics, Chemistry and Biology, Linköping University, Linköping, Sweden.
[b] Division of Theoretical Chemistry, Department of Physics, Chemistry and Biology, Linköping University, Linköping, Sweden.
[c] Department of Pathology, University of California San Diego, La Jolla, California 92093-0612, United States
[d]Department of Physics, Norwegian University of Science and Technology,7491 Trondheim, Norway

*Corresponding author; E-mail: petni@ifm.liu.se

## Table of Content

**Supporting Figures and Tables** **S2**

**Experimental Details** **S4**

**References** **S11**

**NMR spectra** **S12**

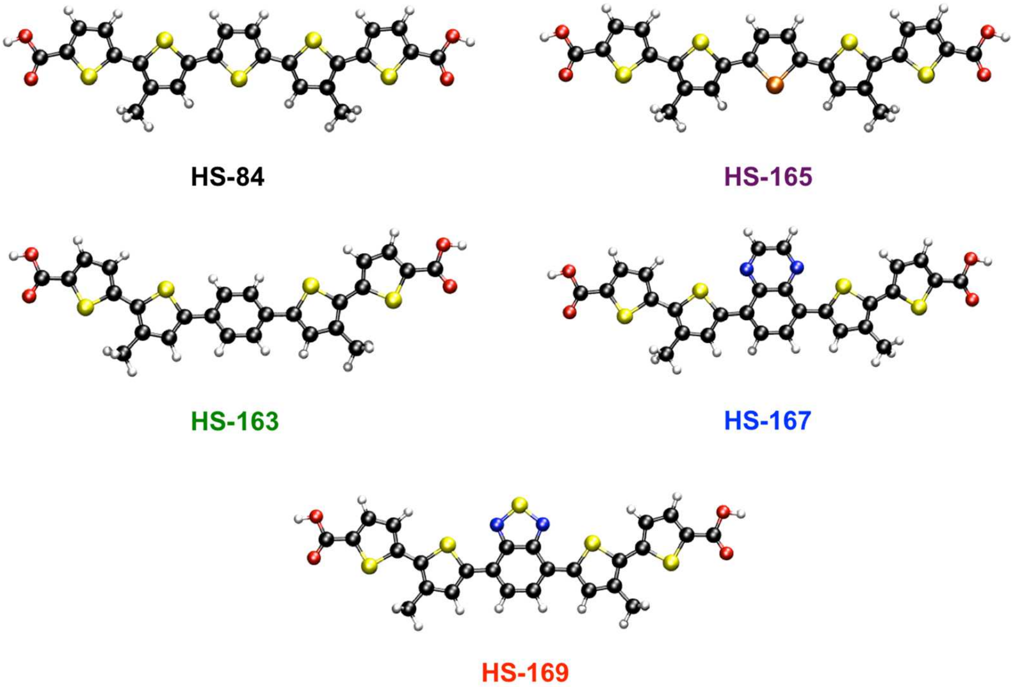


**Figure S1: Model systems used in DFT calculations**

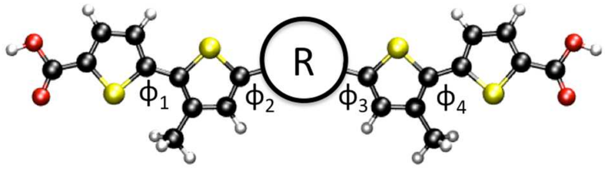


| | $\phi_1$ | $\phi_2$ | $\phi_3$ | $\phi_4$ |
|---|---|---|---|---|
| **HS-84** | 12.8 | 15.9 | 15.9 | 12.8 |
| **HS-165** | 10.5 | 18.3 | 18.3 | 10.5 |
| **HS-163** | 13.3 | 24.1 | 24.1 | 13.3 |
| **HS-167** | 0.0 | 0.1 | 0.1 | 0.0 |
| **HS-169** | 0.9 | 2.9 | 2.9 | 0.9 |

**Figure S2: Inter-ring torsional angles.**

## Table S1: Experimental and calculated main absorption bands, and emission maxima ($Em_{max}$) for the oligomer derivatives

| | Experimental λ [nm] | Theoretical λ [nm] | f | main MO contribution | $Em_{max}$ [nm] | Stoke shift [nm] |
|---|---|---|---|---|---|---|
| **HS-84** | 425 | 445 | 1.925 | H->L (67%) | 535 | 110 |
| **HS-163** | 395 | 400 | 2.003 | H->L (64%) | 492 | 97 |
| **HS-165** | 432 | 450 | 1.902 | H->L (67%) | 545 | 113 |
| **HS-167** | 463 | 491 | 1.446 | H->L (68%) | 690 | 199 |
| | 356 | 344 | 0.667 | H->L+2 (62%) / H-1 -> L+1 (27%) | 690 | 334 |
| **HS-169** | 501 | 531 | 1.311 | H->L (69%) | 708 | 207 |
| | 362 | 354 | 0.804 | H->L+2 (62%) / H-1 -> L+1 (27%) | 708 | 346 |

## Table S2: Optical properties of the oligomer derivatives in PBS or bound to recombinant Aβ 1-42 amyloid fibrils and protein aggregates in tissue samples

| | PBS $Exc_{max}$ [nm] | PBS $Em_{max}$ [nm] | Aβ 1-42 fibrils $Exc_{max}$ [nm] | Aβ 1-42 fibrils $Em_{max}$ [nm] | Aβ deposits $Em_{max}$ [nm] | Tau deposits $Em_{max}$ [nm] |
|---|---|---|---|---|---|---|
| **HS-84** | 425 | 535 | 430, 456, 485 | 504, 539 | 512, 547 | 512, 547 |
| **HS-163** | 395 | 492 | 415 | 468, 492 | 468, 494 | 468, 494 |
| **HS-165** | 432 | 545 | 436, 464, 493 | 515, 552 | 520, 556 | 520, 556 |
| **HS-167** | 363, 470[a] | 690[a] | 375, 510 | 635 | 626 | 626 |
| **HS-169** | 363, 490[a] | 708[a] | 377, 536 | 665 | 661 | 661 |

[a] Determined from a solution containing 10 μM of the oligomer derivative. For the rest of experiment, 300 nM of the oligomer derivatives were used.

# Experimental Details

## *General methods*

NMR spectra were recorded on a Varian 300 instrument (Varian Inc., Santa Clara, CA, USA) operating at 300 MHz for 1H and 75.4 MHz for 13C, using the residual solvent signal as reference. Chemicals and solvents were obtained from commercial sources and used as received. TLC was carried out on Merck pre coated 60 $F_{254}$ plates using UV-light (λ = 254 nm and 366 nm) and charring with ethanol/sulfuric acid/*p*-anisaldehyde/acetic acid 90:3:2:1 for visualization. MALDI-TOF spectra were recorded on a Voyager-DE STR Biospectrometry Workstation using a-cyano-4-hydroxycinnamic acid as a matrix and reference.

## *Synthesis of the thiophene based pentameric ligands*

The synthesis of HS-84 has been published elsewhere[1]. HS-163, HS-165, HS-167 and HS-169 were synthesized as outlined below.

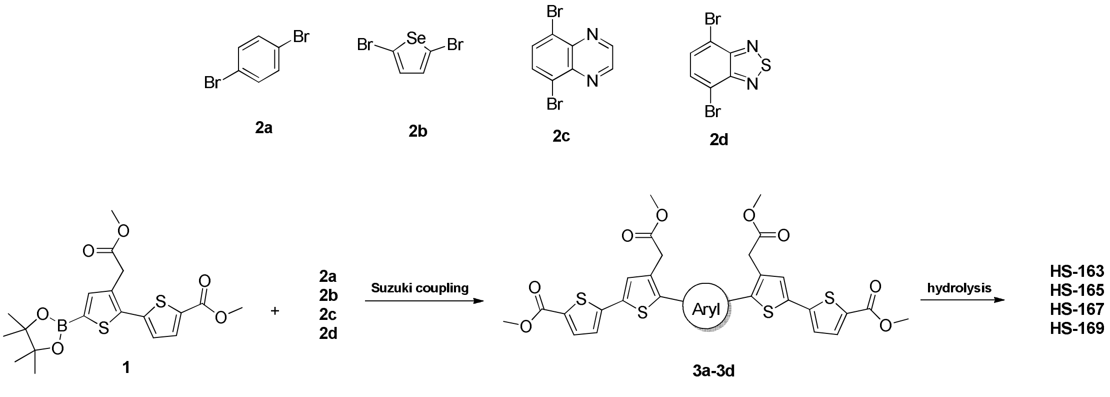


***Scheme 1.*** **Schematic presentation of the general synthetic route for HS-163, HS-165, HS-167 and HS-169**

## *General procedure for Suzuki coupling*

PEPPS-IPr (5 mol %) was added to a mixture of the bromo thiophene derivatives (1-2 equiv), $K_2CO_3$ (3 equiv./bromine), the dimer **1**[2] (2 equiv./aryl) in 1,4-dioxane/methanol

(8 : 2, 8 mL/mmol, degassed). The mixture was heated to 70 °C for 20 min then cooled to room temperature (RT) and pH adjusted to 4 by 1 M HCl. The residue was extracted with DCM (3×30 mL/mmol), washed with water (3×30 mL/mmol), brine (30 mL) and the combined organic phase was dried over $MgSO_4$. The crude product was treated with appropriate solvent to give desired products.

***General procedure for methylesters hydrolysis***

NaOH (1 M, 1.5 equiv./ester) was added to a solution of the oligothiophene in 1,4-dioxane-$H_2O$ (6:1) (7 mL/1 mmol) and heated to 60 °C for 16 h. More water was added and the solution was lyophilized.

***Dimethyl 5',5'''-(1,4-phenylene)bis(4'-(2-methoxy-2-oxoethyl)-[2,2'-bithiophene]-5-carboxylate) (3a)***

General procedure of Suzuki coupling was applied starting with dimer **1**[2] (118 mg, 0.280 mmol) and **2a** (30 mg, 0.127 mmol). The crude product was treated with warm MeOH to give pentamer **3a** (48 mg, 82 %) as yellow solid.

$^1$H NMR (300 MHz, $CDCl_3$) δ 7.77 (d, *J* = 3.9 Hz, 1H), 7.61 (s, 2H), 7.32 (s, 1H), 7.22 (d, *J* = 3.9 Hz, 1H), 3.91 (s, 3H), 3.82 (s, 2H), 3.76 (s, 3H). $^{13}$C NMR (75 MHz, $CDCl_3$) δ 171.0, 162.6, 143.4, 142.1, 134.2, 133.3, 133.2, 132.5, 132.1, 127.1, 126.9, 126.3, 52.5, 52.4, 35.1. MALDITOF: *m/z* calcd for $C_{32}H_{26}O_8S_4$ $(M+H)^+$: 667.1. Found: 667.3.

***Dimethyl 5',5'''-(selenophene-2,5-diyl)bis(4'-(2-methoxy-2-oxoethyl)-[2,2'-bithiophene]-5-carboxylate) (3b)***

General procedure of Suzuki coupling was applied starting with dimer **1**[2] (77 mg, 0.182 mmol) and **2b** (25 mg, 0.87 mmol). The crude product was treated with warm MeOH to give pentamer **3b** (44 mg, 71 %) as red solid.

$^{1}$H NMR (300 MHz, $CDCl_3$) δ 7.76 (d, *J* = 3.9 Hz, 1H), 7.26 (s, 1H), 7.20 (d, *J* = 3.9 Hz, 1H), 7.08 (s, 1H), 3.91 (s, 3H), 3.78 (s, 2H), 3.76 (s, 3H). $^{13}$C NMR (75 MHz, $CDCl_3$) δ 170.7, 162.3, 141.5, 140.6, 138.8, 134.1, 133.1, 132.1, 131.5, 127.7, 127.2, 126.9, 52.4, 52.3, 34.8. MALDITOF: *m/z* calcd for $C_{30}H_{24}O_8S_4Se$ $(M+H)^+$: 721.0. Found: 721.0.

***Dimethyl 5',5'''-(quinoxaline-5,8-diyl)bis(4'-(2-methoxy-2-oxoethyl)-[2,2'-bithiophene]-5-carboxylate) (3c)***

General procedure of Suzuki coupling was applied starting with dimer **1**[2] (130 mg, 0.306 mmol) and **2c** (40 mg, 0.139 mmol). The crude product was treated with warm MeOH to give pentamer **3c** (80 mg, 80 %) as red solid.

$^{1}$H NMR (300 MHz, $CDCl_3$) δ 9.01 (s, 1H), 8.19 (s, 1H), 7.82 – 7.78 (m, 2H), 7.30 (d, *J* = 3.9 Hz, 1H), 3.92 (s, 3H), 3.89 (s, 2H), 3.77 (s, 3H). $^{13}$C NMR (75 MHz, $CDCl_3$) δ 170.3, 161.6, 142.7, 141.5, 138.7, 136.9, 135.1, 133.3, 132.2, 130.5, 130.0, 129.2, 126.2, 126.0, 51.5, 51.4, 34.0. MALDITOF: *m/z* calcd for $C_{34}H_{26}N_2O_8S_4$ $(M+H)^+$: 719.1. Found: 719.1.

***Dimethyl 5',5'''-(benzo[c][1,2,5]thiadiazole-4,7-diyl)bis(4'-(2-methoxy-2-oxoethyl)-[2,2'-bithiophene]-5-carboxylate) (3d)***

General procedure of Suzuki coupling was applied starting with dimer **1**[2] (115 mg, 0.272 mmol) and **2d** (40 mg, 0.136 mmol). The crude product was treated with warm MeOH to give pentamer **3d** (76 mg, 77 %) as red solid.

$^1$H NMR (300 MHz, $CDCl_3$) δ 8.08 (s, 1H), 7.88 (s, 1H), 7.80 (d, *J* = 3.9 Hz, 1H), 7.29 (d, *J* = 3.9 Hz, 1H), 3.92 (s, 3H), 3.90 (s, 2H), 3.78 (s, 3H). $^{13}$C NMR (75 MHz, $cdcl_3$) δ 170.1, 161.6, 151.5, 141.0, 137.8, 133.3, 133.2, 132.4, 131.3, 130.0, 126.3, 124.7, 124.5, 51.6, 51.5, 34.1. MALDITOF: *m/z* calcd for $C_{32}H_{24}O_8S_5$ $(M+H)^+$: 725.0. Found: 725.

***HS-163***

General procedure of ester hydrolysis was performed starting with **3a** (18 mg, 27 µmol). The HS-163 salt was obtained quantitatively as yellow solid.

$^1$H NMR (300 MHz, $D_2O$) δ 7.67 (s, 2H), 7.50 (d, *J* = 3.9 Hz, 1H), 7.33 (s, 1H), 7.16 (d, *J* = 3.9 Hz, 1H), 3.69 (s, 2H). $^{13}$C NMR (75 MHz, D2O) δ 179.3, 169.6, 141.3, 140.0, 140.0, 135.4, 132.3, 131.4, 131.3, 127.8, 125.8, 125.4, 38.2. MALDITOF: *m/z* calcd for $C_{28}H_{18}N_2O_8S_4$ $(M+H)^+$: 611.0. Found: 611.1.

***HS-165***

General procedure of ester hydrolysis was performed starting with **3b** (25 mg, 35 µmol). The HS-165 salt was obtained quantitatively as red solid.

$^1$H NMR (300 MHz, $D_2O$) δ 7.47 (dd, *J* = 4, 0.4 Hz, 1H), 7.25 (s, 1H), 7.07 (dd, *J* = 4.0, 0.4 Hz, 1H), 7.02 (s, 1H), 3.65 (s, 2H). $^{13}$C NMR (75 MHz, $d_2o$) δ 179.1, 169.5, 140.2, 140.0, 139.7, 137.0, 135.1, 131.3, 131.0, 128.9, 127.0, 125.8, 38.7. MALDITOF: *m/z* calcd for $C_{26}H_{16}O_8S_4Se$ $(M+H)^+$: 665.0. Found: 665.0.

***HS-167***

General procedure of ester hydrolysis was performed starting with **3c** (17 mg, 24 µmol). The HS-167 salt was obtained quantitatively as red solid.

$^1$H NMR (300 MHz, $D_2O$) δ 8.81 (s, 1H), 7.90 (s, 1H), 7.42 – 7.38 (m, 2H), 7.03 (d, *J* = 3.9 Hz, 1H), 3.65 (s, 2H). $^{13}$C NMR (75 MHz, $D_2O$) δ 179.4, 169.6, 143.2, 140.3, 139.6, 138.1, 135.2, 135.1, 133.5, 131.4, 131,0, 129.8, 126.8, 125.4, 38.2. MALDITOF: *m/z* calcd for $C_{30}H_{18}N_2O_8S_4$ $(M+H)^+$: 663.0. Found: 663.0.

***HS-169***

General procedure of ester hydrolysis was performed starting with **3d** (41 mg, 55 µmol). The HS-169 salt was obtained quantitatively as red solid.

$^1$H NMR (300 MHz, $D_2O$) δ 7.66 (s, 1H), 7.60 (s, 1H), 7.46 (d, *J* = 3.9 Hz, 1H), 7.07 (d, *J* = 3.9 Hz, 1H), 3.70 (s, 2H). $^{13}$C NMR (75 MHz, $D_2O$) δ 179.2, 169.6, 151.2, 139.9, 136.1, 134.5, 133.9, 131.4, 130.8, 125.6, 125.0, 123.8, 38.4. MALDITOF: *m/z* calcd for $C_{328}H_{16}N_2O_8S_5$ $(M+H)^+$: 669.0. Found: 669.1.

***Optical characterization of the thiophene based pentameric ligands***

Stock solutions of ligands (1.5 mM) were diluted to 10 µM in phosphate buffered saline (PBS, 10 mM phosphate, 140 mM NaCl, 2.7 mM KCl, pH 7.4). Absorption- and emission spectra of the ligands were collected using a Tecan Saphire[2] microplate reader (Tecan, Männedorf, Switzerland).

***Characterization of the thiophene based pentameric ligands towards recombinant Aβ 1-42 amyloid fibrils***

The fibrillation protocol of Aβ1-42 has been published elsewhere.[3] Briefly, recombinant Aβ1-42 peptide lyophilized in hydroxyfluoroisopropanol (rPeptide, Athens, GA, USA) was dissolved in 2 mM NaOH to a stock concentration of 1 mg/ml. The solution of Aβ1-42 was diluted with PBS (10 mM phosphate, 140 mM NaCl, 2.7 mM KCl, pH 7.4) to a final concentration of 10 µM and added to the wells of a microtiter plate (Corning). The microtiter plate was incubated at 37°C in quiescent mode for 48 hours and the presence of recombinant Aβ1-42 amyloid fibrils was confirmed by thioflavin-T (ThT) staining. 300 nM of the respective thiophene based pentameric ligand was added to 10 µM fibrils or PBS (10 mM phosphate, 140 mM NaCl, 2.7 mM KCl, pH 7.4) and the excitation- and emission spectra for each probe were collected using a Tecan Saphire[2] microplate reader (Tecan, Männedorf, Switzerland).

***Staining and spectral analysis of histological samples***

Frozen brain sections (10 µm) from brain tissues from clinically and neuropathologically well-characterized cases of AD were fixed in 96% EtOH,

rehydrated in 50% EtOH and $dH_2O$ and then incubated in phosphate buffered saline (PBS, 10 mM phosphate, 140 mM NaCl, 2.7 mM KCl, pH 7.4) for 10 min. HS-84, HS-163, HS-165, HS-167 or HS-169 were diluted to 600 nM in PBS and added to the sections. After 30 min, the sections were washed with PBS and mounted with Dako fluorescent mounting medium (Dako Cytomation, Glostrup, Denmark). The mounting medium was allowed to solidify over night before collecting emission spectra of the ligands bound to misfolded Aβ and tau using an inverted LSM 780 confocal microscope (Carl Zeiss, Oberkochen, Germany) with excitation wavelength at 405 nm (HS-163), 458 nm (HS-84 and HS-165), 514 nm (HS-167) or 534 nm (HS-169). For the tissue samples, all subjects provided informed consent for research participation and for brain donation after death under an IRB-approved protocol.

***Computational Details***

All calculations were performed with use of the Gaussian program.[4] Ground state geometry optimization was performed using the B3LYP[5] functional in combination with the 6-31+G(d) basis set.[6] For TD-DFT calculations, the same basis set was used in combination with the long-range corrected functional CAM-B3LYP[7] in order to correctly take into account the charge transfer character that is inherent in any donor–acceptor systems. The vertical excitation energies and oscillator strengths were calculated for the 10 first singlet excited states and a Gaussian broadening with a half-width at half maximum of 0.3 eV was used to plot the final UV absorption spectra.

## *References*


1 R. A. Simon, H. Shirani, K. O. A. Åslund, M. Bäck, V. Haroutunian, S. Gandy, K. P. R. Nilsson, *Chemistry* 2014, **20**, 12537.

2 T. Klingstedt, H. Shirani, J. Mahler, B.M. Wegenast-Braun, S. Nyström, M. Goedert, M. Jucker, K.P.R. Nilsson, *Chemistry* 2015, **21**, 9072.

3 T. Klingstedt, A. Åslund, R. A. Simon, L. B. G. Johansson, J. J. Mason, S. Nyström, P. Hammarström and K. P. R. Nilsson, *Org. Biomol. Chem.* 2011, **9**, 8356.

4 M. J. Frisch, G. W. Trucks, H. B. Schlegel, G. E. Scuseria, M. A. Robb, J. R. Cheeseman, G. Scalmani, V. Barone, B. Mennucci, G. A. Petersson, H. Nakatsuji, M. Caricato, X. Li, H. P. Hratchian, A. F. Izmaylov, J. Bloino, G. Zheng, J. L. Sonnenberg, M. Hada, M. Ehara, K. Toyota, R. Fukuda, J. Hasegawa, M. Ishida, T. Nakajima, Y. Honda, O. Kitao, H. Nakai, T. Vreven, J. A. Montgomery, J. E. Peralta, F. Ogliaro, M. Bearpark, J. J. Heyd, E. Brothers, K. N. Kudin, V. N. Staroverov, R. Kobayashi, J. Normand, K. Raghavachari, A. Rendell, J. C. Burant, S. S. Iyengar, J. Tomasi, M. Cossi, N. Rega, N. J. Millam, M. Klene, J. E. Knox, J. B. Cross, V. Bakken, C. Adamo, J. Jaramillo, R. Gomperts, R. E. Stratmann, O. Yazyev, A. J. Austin, R. Cammi, C. Pomelli, J. W. Ochterski, R. L. Martin, K. Morokuma, V. G. Zakrzewski, G. A. Voth, P. Salvador, J. J. Dannenberg, S. Dapprich, A .D. Daniels, Ö. Farkas, J. B. Foresman, J. V. Ortiz, J. Cioslowski and D. J. Fox, Gaussian 09, Revision A.02, Gaussian, Inc., Wallingford, CT, 2004.

5 (a) A.D. Becke, *J. Chem. Phys.*, 1993, **98**, 5648; (b) C. Lee, W. Yang, R. G. Parr, *Phys. Rev. B*, 1988, **37**, 785; (c) S. H. Vosko, L. Wilk, M. Nusair, *Can. J. Phys.*, 1980, **58**, 1200; (d) P. J. Stephens, F. J. Devlin, C. F. Chabalowski, M. J. Frisch, *J. Phys. Chem.*, 1994, **98**, 11623.

6 (a) W. J. Hehre, R. Ditchfield and J. A. Pople, *J. Chem. Phys.*, 1972, **56**, 2257; (b) P. C. Hariharan and J. A. Pople, *Theor. Chim. Acta*, 1973, **28**, 213.

7 T. Yanai, D. P. Tew and N. C. Handy, *Chem. Phys. Lett.*, 2004, **393**, 51.

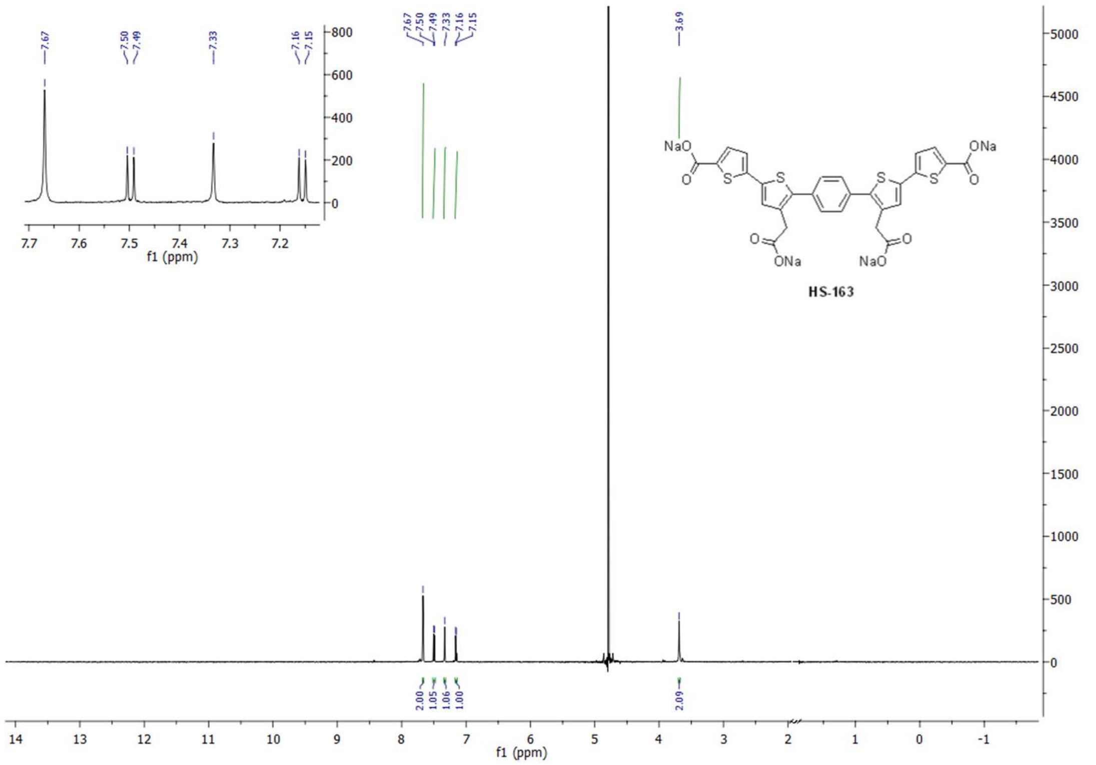
HS-163
f1 (ppm)
3.69
7.67
7.50
7.49
7.33
7.16
7.15
2.00
1.05
1.06
1.00
2.09

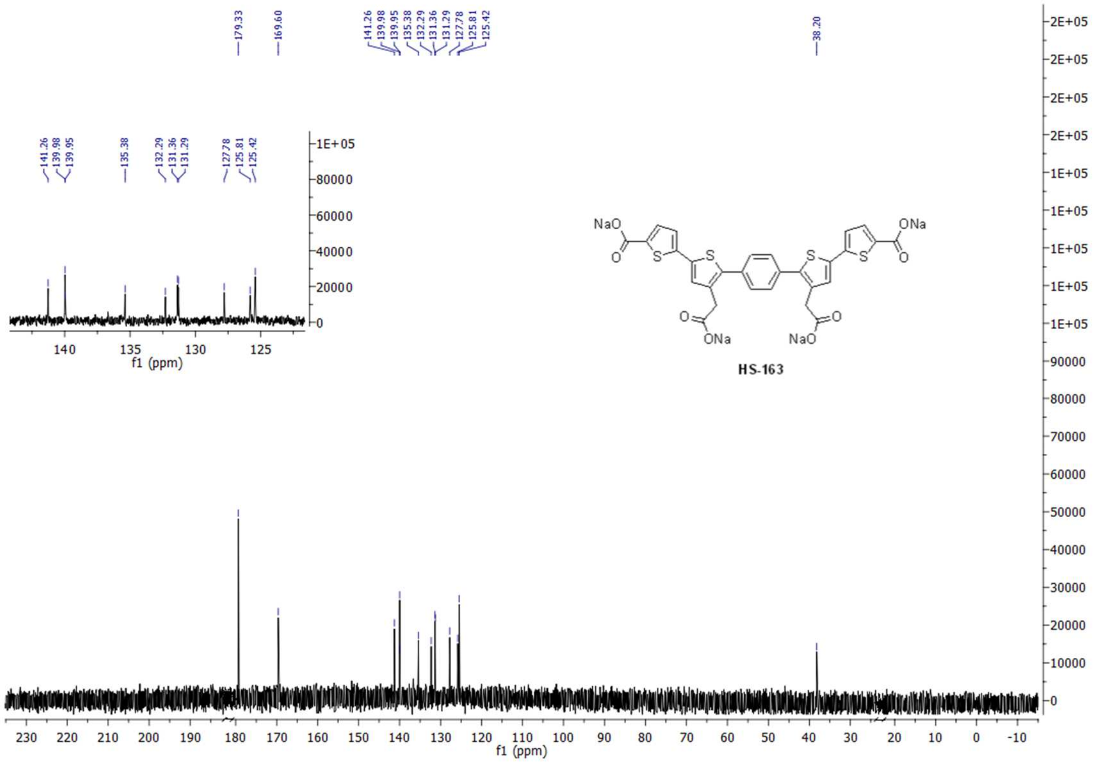
HS-163
f1 (ppm)
179.33
169.60
141.26
139.98
139.95
135.38
132.29
131.36
131.29
127.78
125.81
125.42
38.20

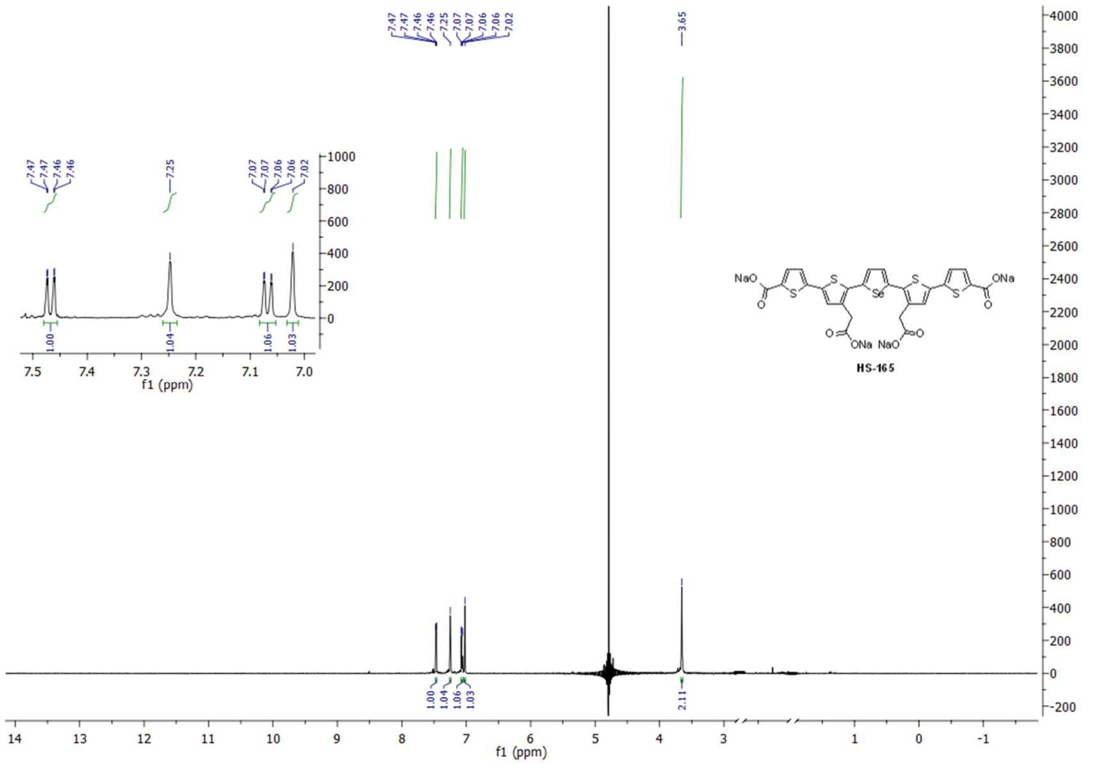
7.47
7.47
7.46
7.46
7.25
7.07
7.07
7.06
7.06
7.02
3.65
1.00
1.04
1.06
1.03
2.11
f1 (ppm)
NaO
ONa
Se
HS-165

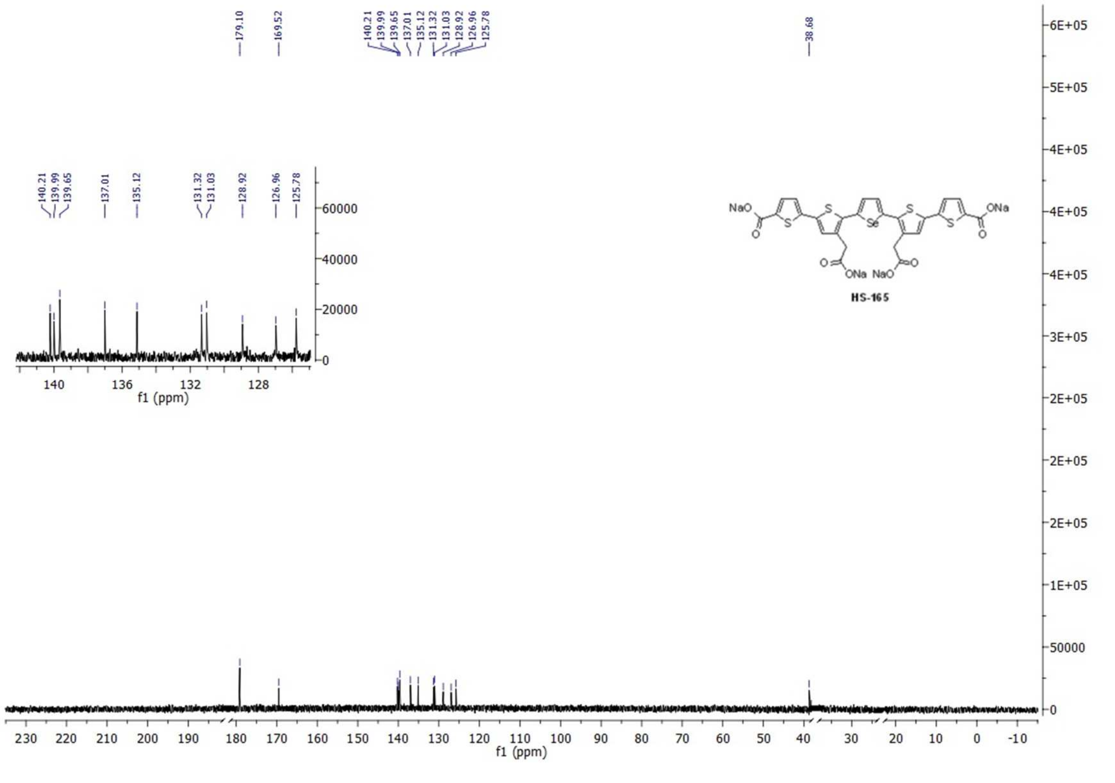
179.10
169.52
140.21
139.99
139.65
137.01
135.12
131.32
131.03
128.92
126.96
125.78
38.68
f1 (ppm)
NaO
ONa
Se
HS-165

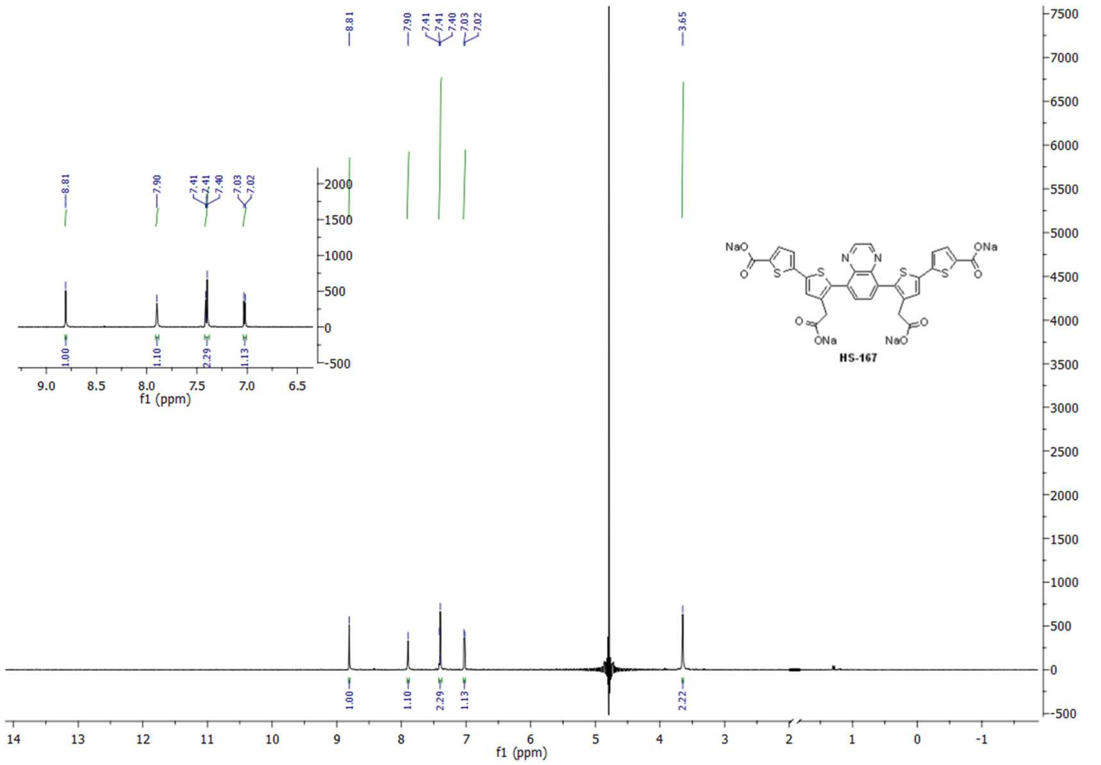
8.81
7.90
7.41
7.41
7.40
7.03
7.02
3.65
1.00
1.10
2.29
1.13
2.22
f1 (ppm)
NaO
ONa
N
S
HS-167

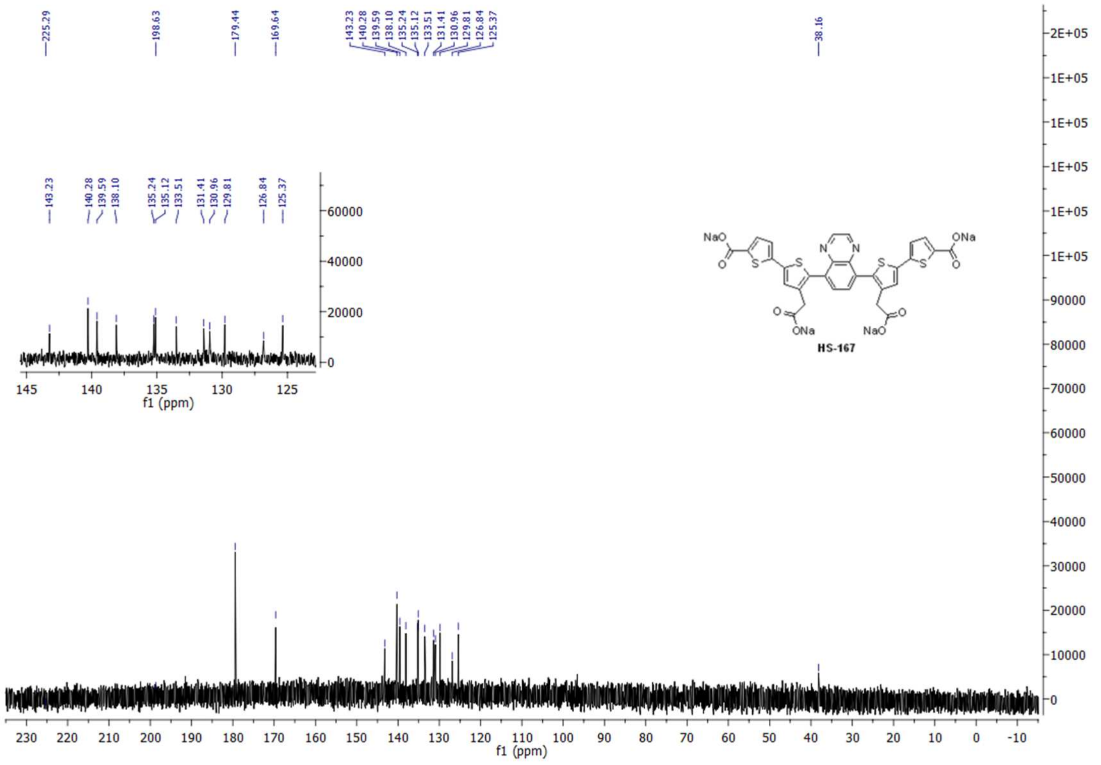
225.29
198.63
179.44
169.64
143.23
140.28
139.59
138.10
135.24
135.12
133.51
131.41
130.96
129.81
126.84
125.37
38.16
f1 (ppm)
NaO
ONa
N
S
HS-167

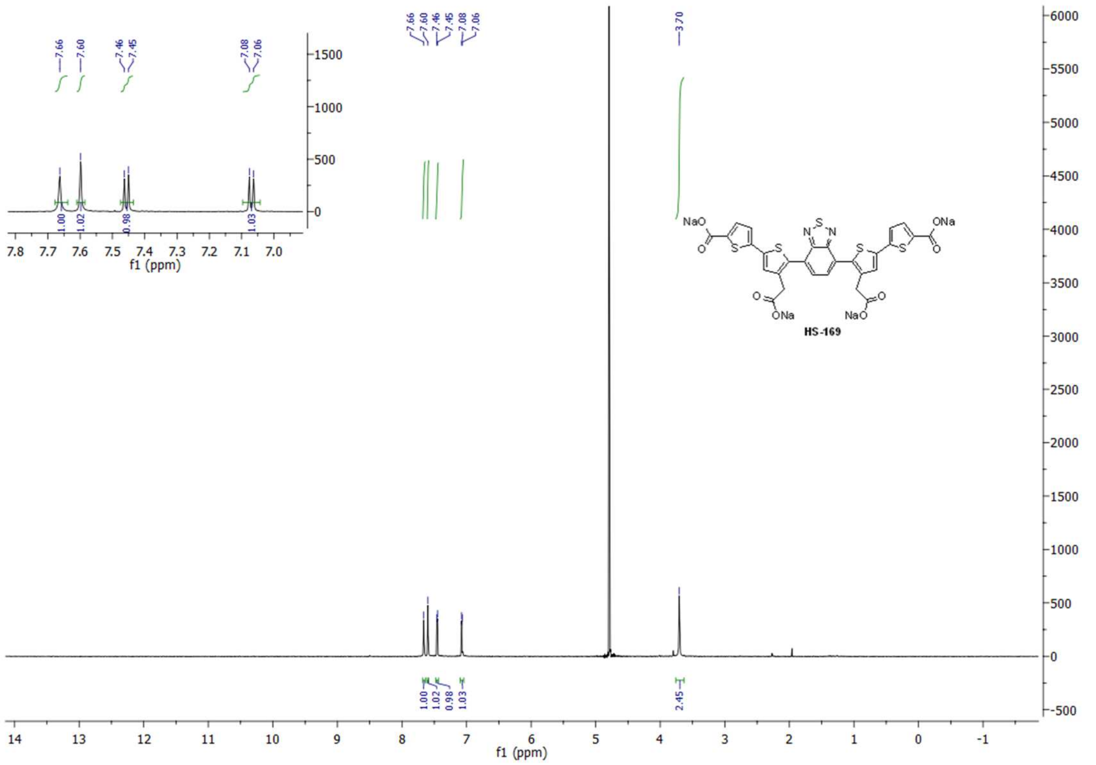
7.66
7.60
7.46
7.45
7.08
7.06
3.70
1.00
1.02
0.98
1.03
2.45
f1 (ppm)
HS-169

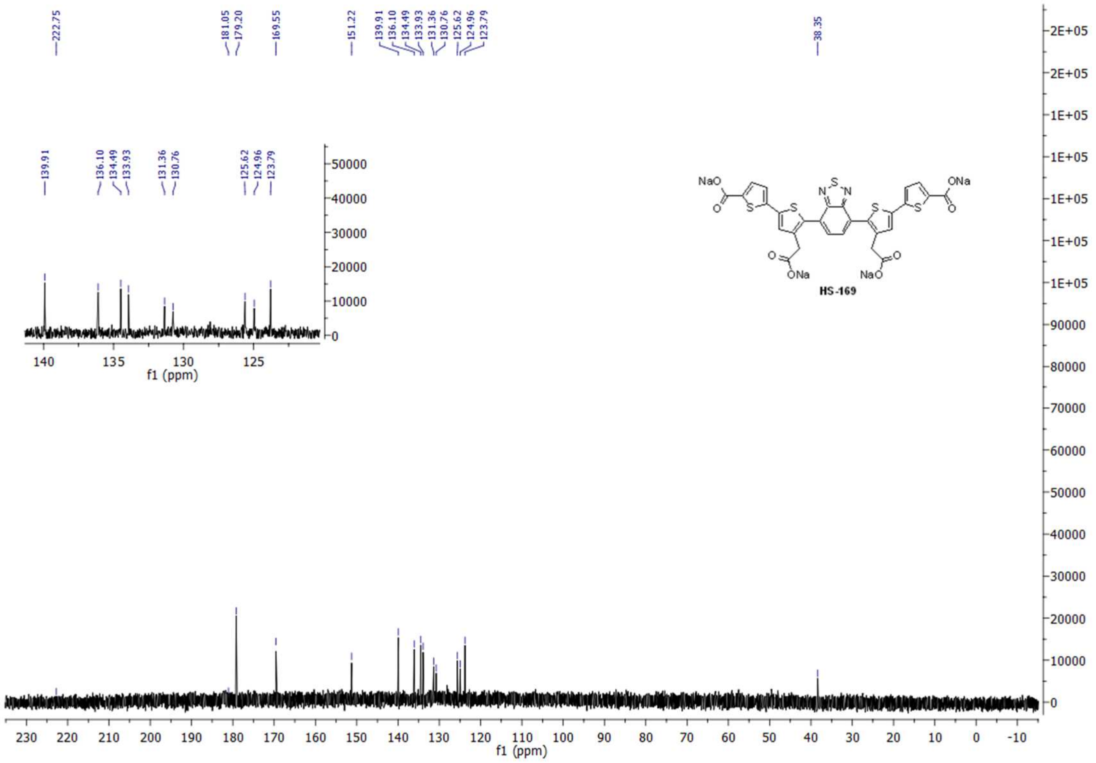
222.75
181.05
179.20
169.55
151.22
139.91
136.10
134.49
133.93
131.36
130.76
125.62
124.96
123.79
38.35
f1 (ppm)
HS-169
